# Decreased Nocturnal Awakenings in Young Adults Performing Bikram Yoga: A Low-Constraint Home Sleep Monitoring Study

**DOI:** 10.5402/2012/153745

**Published:** 2012-04-12

**Authors:** Ravi S. Kudesia, Matt T. Bianchi

**Affiliations:** ^1^Sleep Division, Neurology Department, Massachusetts General Hospital, Wang 720, Boston, MA 02114, USA; ^2^Olin Business School, Washington University in St. Louis, Campus Box 1133, One Brookings Dr., St. Louis, MO 63130, USA

## Abstract

This pilot study evaluated the impact of Bikram Yoga on subjective and objective sleep parameters. We compared subjective (diary) and objective (headband sleep monitor) sleep measures on yoga versus nonyoga days during a 14-day period. Subjects (*n* = 13) were not constrained regarding yoga-practice days, other exercise, caffeine, alcohol, or naps. These activities did not segregate by choice of yoga days. Standard sleep metrics were unaffected by yoga, including sleep latency, total sleep time, and percentage of time spent in rapid eye movement (REM), light non-REM, deep non-REM, or wake after sleep onset (WASO). Consistent with prior work, transition probability analysis was a more sensitive index of sleep architecture changes than standard metrics. Specifically, Bikram Yoga was associated with significantly faster return to sleep after nocturnal awakenings. We conclude that objective home sleep monitoring is feasible in a low-constraint, real-world study design. Further studies on patients with insomnia will determine whether the results generalize or not.

## 1. Introduction

 Yoga is a technique of spiritual development originating in Ancient India which was first documented in the Yoga Sutras of Patanjali, written around 500–100 BC [1]. It describes eight essential limbs, two of which are stretching and breathing exercises. Bikram Yoga is a modern style introduced in the 1970s by Bikram Choudhury that is more focused on creating physical wellness than spiritual development. It utilizes a specific sequence of 26 stretching postures and 2 breathing exercises, which are performed over a 90 minutes session at 105 degrees Fahrenheit and 40% humidity. Because the Bikram Yoga series and environment are so highly regimented and common to all facilities, it provides an important opportunity to study the effects of physical yoga on subjective and objective metrics of sleep. In particular, anecdotal evidence of a relationship between Bikram practice and quality of sleep has circulated for some time within communities of practitioners, but this has not been formally studied.

Insomnia is a condition of difficulty initiating or maintaining sleep that affects 5–40% of adults at some point in their lives and thus represents a major concern for health and wellbeing [2, 3]. Approximately 10% of adults report their insomnia to be chronic and/or severe. Many individuals self-medicate with over-the-counter and complementary remedies [4]. Prescription hypnotics may also be used but are associated with some liabilities including potential for tolerance or dependence, among other side effects [5]. Nonpharmacological means to improve sleep, such as cognitive behavioral therapy, have been shown to be at least as effective as pharmacological therapies [6]. A recent meta-analysis of alternative therapies included methods spanning natural remedies, yoga, acupuncture/acupressure, and meditation [7]. Only one out of the 20 studies that met criteria for inclusion in that meta-analysis reported objective sleep measurements. Emphasis on subjective report in the literature may derive in part from the diagnostic criteria for insomnia, which do not require objective metrics. Complementing subjective report with objective home sleep monitoring may prove to be an important component of comprehensive sleep disorder management.

Several major challenges in sleep research may be addressed by objective home sleep monitoring. One is that while the sleep laboratory setting allows highly controlled and mechanistic investigation of insomnia (among other sleep disorders), this advantage comes at the expense of uncertain external validity: a single night in an unnatural environment may not capture contributions by the diversity of behaviors and exposures affecting sleep in day-to-day life. This has been demonstrated in other species, as mice exhibit markedly different activity rhythms “in the field” compared to laboratory conditions [8]. Another is that the routinely employed methods used to analyze sleep architecture have been shown to be insensitive for detecting differences, especially those related to fragmentation. For example, the fragmentation associated with sleep apnea is not well appreciated using stage percentage and sleep efficiency metrics but can easily be seen with probabilistic metrics such as the distribution of bout durations [9–11]. These metrics are best estimated by repeated measures within individuals. Given the variability found in patients with insomnia in terms of severity, contributing factors, and impact on health and well being, it is becoming increasingly important to find personalized solutions, ideally while concurrently minimizing the need for hypnotic agents.

We performed a low-constraint, observational study of sleep architecture in healthy young adults over a 14-day period in which they performed Bikram Yoga on at least two of the days. Subjective sleep was assessed with a daily diary. Objective sleep was assessed with a commercially available headband monitoring device worn at night. Subjects were allowed to self-determine the days and times of Bikram and how many sessions to perform (with a minimum of two days). They were also unconstrained as to engaging in other exercise, napping, and consumption of alcohol, nicotine, and caffeine. Such low-constraint designs are not often adopted in clinical research because the confounding (uncontrolled) variables may correlate with the intervention of interest; however, highly controlled studies suffer from the inverse problem: questionable relevance to real-world implementation. We undertook this pilot study with the following aims: (1) to investigate the feasibility of using a simple home monitor to characterize sleep architecture in a low-constraint setting; and (2), to determine if Bikram Yoga acutely affects the sleep architecture of healthy young adults.

## 2. Methods

### 2.1. Subject Population

Institutional Review Board approval was obtained to conduct this study through the Massachusetts General Hospital. The inclusion criteria included healthy adults aged 18–45 who reported that they either perform Bikram Yoga or were planning to try it. Subjects were instructed to perform Bikram yoga on at least two days during the recording period (and no more than 12 days). The flexibility was meant to mimic “real world” decisions, along with the subjects having the choice of day and time to perform the Bikram and other activities reported on the diary (nap, exercise, caffeine, alcohol, and nicotine). Subjects performed Bikram Yoga at local providers in the Boston/Cambridge area. Subjects were compensated for participation. The exclusion criteria included medical problems or medications that could potentially interfere with the sleep monitoring device's algorithm (such as neuroactive medications that affect the EEG and/or eye movements, epilepsy or skull defects that could result in breach confounds), significant neurological disease, or any known sleep disorder. Twenty subjects consented, and 13 completed the study. One subject withdrew because of inability to sleep with the monitoring device (discomfort); one was excluded for failure to return the diary; one was excluded for inaccuracy of sleep scoring attributed to severe bruxism (large blocks of wake reported in the night, despite subject denying nocturnal awakenings, and these were presumed to reflect muscle artifact leading to excess wake scoring); two were excluded for insufficient objective data (due to headband frequently falling off during sleep); and two did not report reasons for noncompletion.

### 2.2. Sleep Monitoring

Subjects monitored their sleep-wake activity through self-report and through the use of an elastic-headband sleep-monitoring device, the Zeo (http://www.myzeo.com/sleep/). This study was not supported by Zeo; refurbished devices were purchased from Zeo Inc. (Newton, MA, USA). Subjects were not provided serial numbers to log into the Zeo web site, but they could view their sleep-stage architecture (hypnogram), which was displayed each morning on the alarm-clock display. The headband contains embedded fabric sensors to enable “dry” contact with the forehead and does not require special skin preparation or adhesive. A neural network algorithm uses frontal electroencephalographic signals, electromyographic signals, and electrooculographic signals to classify sleep-wake stages according to the following categories: wake, rapid eye movement (REM) sleep, light non-REM, and deep non-REM sleep. Light non-REM corresponds to N1 and N2 stages, while deep non-REM corresponds to stage N3. Correlation of Zeo classification of sleep architecture with human scoring is ~80% [12, 13]. The raw data is not stored; rather, the device stores the hypnogram data only, which is then exported for analysis. The written diary enabled self-report recording of the following parameters each day: alcohol, caffeine, tobacco, naps, exercise, Bikram, subjective sleep latency, total sleep time, and morning Stanford Sleepiness Scale.

### 2.3. Analysis

We predefined two types of analysis to perform on the sleep-architecture data (using Prism software, GraphPad software Inc.). First, we summarized sleep using the traditional metrics of sleep latency, total sleep time, and percentage of time spent in each sleep-wake stage, as reported by the device. The distribution of data in this manner (stage percentage) approximated the Gaussian assumption by formal distribution testing (using three different tests of normality: Kolmogorov-Smirnov, D'Agostino-Pearson, and Shapiro-Wilk), and thus we used ANOVA with post hoc testing for multiple comparisons where appropriate.

Second, we considered sleep architecture according to the distribution of sleep-wake-bout durations. Zeo allows export of sleep-stage classification data in 30-second intervals (although some smoothing of brief events occurs in the algorithm). Bout durations were calculated from this data and plotted as “survival curves,” in which the durations of each sleep-wake stage were assessed for each individual based on whether Bikram Yoga was performed during the day or not. The frequency of observations was normalized within Bikram versus non-Bikram days, separately for each patient. This normalization addresses the fact that each subject had a different number of bouts of sleep-wake stages each night and a different number of days on which Bikram was performed. These normalized data were then averaged over the whole group of subjects, to create average survival curves shown in the figures.

The averaged survival curves were analyzed by fitting two-exponential decay curves using the nonlinear sum-of-squares *F*-test to determine whether the time constants and proportions were different in Bikram versus non-Bikram conditions. The nonlinear sum-of-squares *F*-test was also used to establish that a two-exponential decay function was a significantly better fit than a monoexponential function, consistent with our prior results that sleep-wake bout distributions required the sum of multiple exponential functions [9], and that resolving the longest (slowest) time constant required a large amount of data (more than obtained here, e.g.). As an auxiliary test to compare distributions between Bikram and non-Bikram nights, we performed analysis of the integral of the survival curves, which does not require any assumptions about the shape or fitting of the curve, and this showed a significant difference in the WASO survival curves in Bikram versus non-Bikram conditions (*P* < 0.02, *t*-test).

## 3. Results

### 3.1. Subject Characteristics

Table 1 shows the baseline characteristics of the 13 subjects who completed the study. The median number of days of usable data in this group was 13 and the median number of days in which Bikram Yoga was performed was four. The baseline scores were in the normal range for the Epworth Sleepiness Scale (ESS) (normal is ≤10) [14], and the Horne and Ostberg chronotype scale [15]. The Pittsburgh Sleep Quality Index (PSQI) scores were more variable, with a median value of 8 (<5 is considered to indicate good sleep) [16].

### 3.2. Sleep Latency and Total Sleep Time

**Figure 1:**
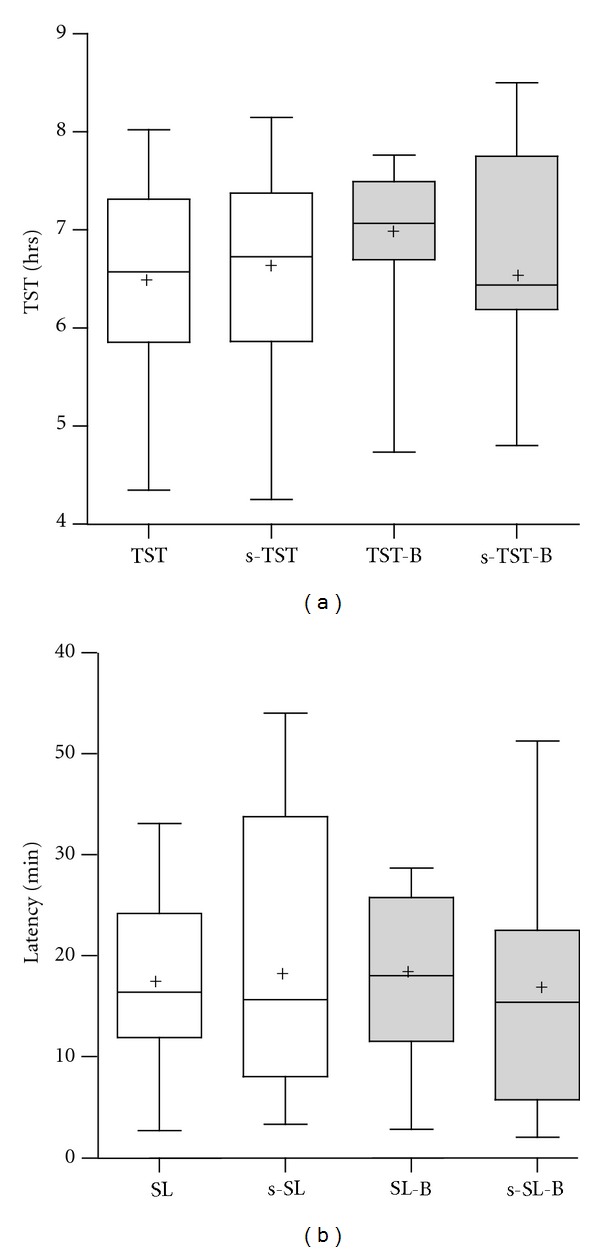
Total sleep time and sleep latency. (a) Total sleep time is shown by objective measurement (TST) and by subjective diary (s-TST) on nonyoga nights (open boxes) and on Bikram Yoga nights (gray boxes). Each plot shows the median, 25% and 75% quartiles (boxes), mean (plus sign), and 95% confidence range (whiskers). There were no significant differences in subjective or objective TST on yoga or nonyoga days. (b) Sleep latency is shown by objective measurement (SL) and by subjective diary (s-SL) on nonyoga nights (open boxes) and on Bikram Yoga nights (gray boxes). Box and whisker plots are as in (a). There were no significant differences in subjective or objective latency on yoga or nonyoga days.

Figure 1 compares Bikram (gray) versus non-Bikram days (white), with regard to objective and subjective measures of total sleep time (Figure 1(a)) and sleep latency (Figure 1(b)). The subjective and objective measures were not significantly different, indicating absence of sleep-state misperception [17] in this healthy population. Performing Bikram Yoga was not associated with significant changes in either subjective or objective measures of sleep latency or total sleep time (ANOVA, with Bonferroni correction, *P* > 0.05).

### 3.3. Sleep Architecture Analysis

**Figure 2:**
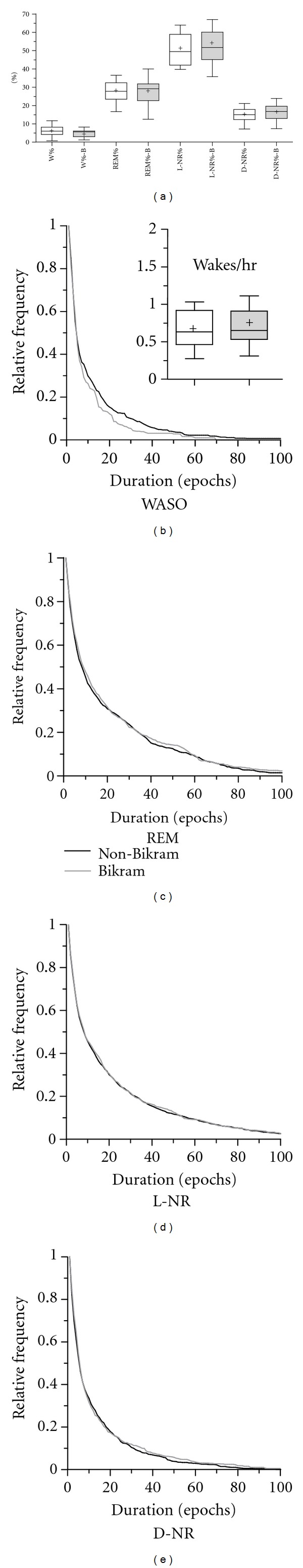
Sleep-wake architecture. (a) The percentage of time spent in wake after sleep onset (W), REM, light NR (L-NR), and deep NR (D-NR) sleep are shown in box and whisker plots (median, 25–75% quartiles, and 95% confidence interval whiskers; mean indicated by plus sign). The values of each sleep-wake stage for nonyoga nights (open boxes) were not different than nonyoga nights (gray boxes). (b) Survival curves for bouts of wake after sleep onset (WASO) were significantly different for nonyoga (black line) and yoga (gray line) nights. The survival curves show the normalized relative frequency of observing bouts of WASO of different durations. The inset shows the absolute number of awakenings per hour of sleep for nonyoga (open) and yoga (gray) nights, which were not different. (c–e) There were no differences in the survival curves between yoga and nonyoga nights for REM, L-NR, or D-NR sleep-stage-bout distributions.

Standard analysis of sleep architecture involves calculating the percentage of time spent in each sleep-wake stage. In this study, we found that the percentage of time spent in wake, light non-REM, deep non-REM, or REM sleep did not differ based on Bikram performance (ANOVA, with Bonferroni correction, *P* > 0.05) (Figure 2(a)). This was not surprising, given that (1) this is a baseline healthy population without significant sleep complaints and (2) sleep-stage percentage is an insensitive metric of fragmentation, such as that caused by sleep apnea [9–11].

Analysis of sleep stages from a probabilistic standpoint is a valuable alternative. Sleep architecture is characterized by transitions between wake and sleep as well as by transitions within sleep among the different stages. A “bout” duration is defined as the amount of consecutive time spent in any given stage of sleep (or wake) before transitioning to the next stage. The distribution of bout durations yields insight into the stability of each sleep-wake stage. Here, we use a type of “survival” analysis, which considers the probability of a bout of sleep or wake lasting a certain length of time (or longer).

Figure 2(b) illustrates the distribution of wake-bout durations in a survival plot. The downward-sloping curve indicates that the probability of observing brief duration awakenings is high, but that of observing progressively longer duration of wake bouts progressively decreases. Our results show that the curve is significantly accelerated on Bikram Yoga days compared to non-Bikram days. We quantified these distributions in two ways, both of which support the conclusion of shorter wake-bout durations with Bikram. The first method involved fitting the distributions with the sum of exponential functions, as we have previously reported [9]. Exponential fitting revealed significantly accelerated survival curve for Bikram days. The time constants for curve fitting of nonyoga days (in minutes, with 95% confidence intervals) were fast tau 1.4 (1.3–1.6), slow tau 10.2 (9.5–10.9), and the percentage of contribution of fast tau 66.4% (63.9–69.0). The time constants for curve fitting of yoga days were fast tau 1.5 (1.3–1.7), slow tau 7.7 (7.0–9.0), and the percentage of contribution of fast tau 67.1% (63.2–70.9), indicating that the majority of the effect of Bikram came in the slow tau, which was 25% faster on yoga days (sum-of-squares *F*-test for parameter difference, *P* < 0.0001). An alternative metric that does not depend on accurate fitting of the distribution involved comparing the area under the survival curve; these values satisfied the Gaussian approximation. This analysis revealed a significant decrease in the area of the WASO distribution comparing Bikram to non-Bikram nights (*P* < 0.03, *t*-test).

The observation that Bikram Yoga was associated with shorter duration awakenings within sleep can be interpreted in two ways: either subjects fell asleep more rapidly following a usual number of nocturnal awakenings or there were in fact additional brief awakenings (perhaps, e.g., via dehydration causing many brief awakenings that would bias the distribution) that dominated the survival-curve analysis. To distinguish these possibilities, we analyzed the number of awakenings per night. The inset of Figure 2(b) shows that the number of awakenings was unchanged (paired *t*-test, *P* > 0.05) and confirms that Bikram Yoga was associated with improved sleep architecture as manifested by more rapid return to sleep for any given nocturnal awakening.

Similar survival analysis was performed for the three sleep substages (Figures 2(c)–2(e)), but no differences were observed using the same time-constant analysis as above. It is worth noting that the reason that the time-constant analysis, but not traditional stage-percentage analysis, can reveal architecture differences is that the percent of time in any stage ignores the bout length: for example, consider two subjects with 90% sleep efficiency—one could have a single 30-minute awakening and the other could have 30 one-minute awakenings. Thus, bout-distribution analysis provides a sensitive tool for comparison of sleep-wake architecture.

## 4. Discussion

This study suggests that home sleep monitors may be an important adjunct to self-report outcomes in longitudinal studies of interventions to improve sleep. Considering the dual factors of objective and subjective sleep, one could assess effects in four potential categories: response in both, no response in both, response to subjective but not objective, and response in objective but not subjective. Each of these dimensions may have distinct clinical and pathophysiological implications. For example, there is evidence for dissociation between objective and subjective sleep durations [18, 19] and evidence that certain medications actually induce a subjective overestimation of sleep duration [20]. Here, we show evidence of the last of these four categories: although we did not observe significant improvements in self-reported sleep quality or quantity, we were able to identify an objective improvement in sleep architecture associated with Bikram Yoga.

### 4.1. Effect of Bikram Yoga on Sleep

Further studies are required to determine which aspect(s) of Bikram Yoga can be most important for impacting sleep physiology. For example, physiological changes could be attributed to changes in hydration, to performance of vigorous exercise in general, or to indirect effects (such as being more mindful about avoiding other disruptors of sleep). We did not detect any significant differences in terms of the portion of days in which subjects reported alcohol, caffeine, naps, or other exercise when comparing Bikram versus non-Bikram days (data not shown). Future studies should compare Bikram to other forms of exercise as well as forms of relaxation, to determine which aspect of Bikram was most relevant to effects on sleep.

In terms of the expectation of finding effects of interventions such as yoga on sleep architecture, it is important to consider differences between the present group (healthy young adults) and other groups with clinically significant sleep disturbance, such as those with insomnia with or without comorbid depression and pain. In healthy cohorts there may be ceiling effects regarding improvements in sleep physiology, which may already be rather optimal compared to those likely to be seen in patient groups. It is possible that other changes would be evident (such as the distribution of sleep substages) in groups performing Bikram Yoga that have more fragmentation at baseline (and thus have more room to change).

### 4.2. Quantifying Sleep Architecture

The manner in which sleep-wake architecture is characterized is critical in order to avoid false-negative findings. There is a long tradition in both clinical and research venues of analyzing sleep architecture according to summary metrics such as sleep efficiency and percentage of time spent in particular sleep-wake stages. However, emerging data suggests that such analysis is insensitive to detect even severe degrees of fragmentation in large cohorts. For example, distinguishing between subjects with severe sleep apnea (who have clear sleep architecture fragmentation) or normal breathing is not accomplished well using sleep-stage percentages but is clearly evident using transition analysis [9–11]. Analyzing distributions provides a powerful method to detect objective changes in architecture, which has important implications for power calculations when planning studies, as well as the interpretation of the objective impact an intervention (such as yoga, or a medication) may have on sleep physiology.

### 4.3. The Potential Advantages of Home Sleep Monitoring

It is clear to patients and investigators alike that sleeping in the laboratory setting may not be reflective of home sleep for two reasons. First, it may be that the extensive wires and sensors in the unusual environment may be disturbing to sleep and/or anxiety provoking. In fact, most laboratory research studies will perform at least one “accommodation” night in hopes of decreasing the untoward effects of the laboratory environment on the measurement of sleep. Second, the true test of efficacy for any intervention regarding sleep is whether the effect can be observed in the home setting, with all of its inherent variability. In addition to these practical measurement issues, it is critical to understand individual differences in sleep habits and sensitivity to factors that potentially enhance or disturb sleep (such as caffeine, alcohol, pain, work, etc.). Thus, there is an important tradeoff to consider: to the extent that one controls these variables in the laboratory environment, one can investigate efficacy and mechanism of interventions; on the other hand, one can perform low-constraint “real world” experiments and allow natural fluctuations in these variable factors, as shown in this study. The latter approach may also be favorable in terms of the opportunity for individualized sleep medicine, where it becomes important to have longitudinal data within subjects to enable investigation of multiple factors that may be relevant to the individual. There is data, for example, suggesting differential sensitivity to sleep deprivation and caffeine [21–23], as well as sleep need [24].

### 4.4. Limitations and Future Directions

In low-constraint studies such as this one, we depend on various factors that potentially influence sleep to be distributed both within individuals (e.g., nights of alcohol use) and between different individuals (e.g., some individuals nap, others do not). However, even if these factors are distributed evenly by chance, their specific occurrences are not prospectively randomized and thus represent potential confounding factors. We chose the low-constraint paradigm to ensure realistic circumstances, but we recognize the possibility that there may be important information contained in how individuals make various behavioral choices. For example, caffeine may be taken to enhance energy after a bad night of sleep. In contrast, consider that restricting or controlling caffeine or alcohol intake may itself be an alteration of one's home-behavioral pattern. Subjects also had a choice, as in “real life,” of when and how often to perform the Bikram Yoga intervention, and we cannot tell whether that choice depended on some unknown factor that was somehow linked to sleep physiology, rather than Bikram Yoga causing the change we observed in WASO.

If Bikram Yoga was causative in the observed WASO improvement, we cannot distinguish which aspect was most important (heat, humidity, time, or poses) or if the total combination was important (perhaps together with other indirect factors). In addition, there may be different acute versus longer-term effects, and these may differ among individuals. Stratified trial designs (which require higher enrollment numbers) that take into account different experience levels, frequency of practice, and acute versus long-term effects, will be important in this regard. Here, we were not powered to stratify by experience. It is possible, for example, that experienced practitioners achieve an improved “chronic” state of sleep physiology that is less sensitive to the acute day-to-day practice (or not) of yoga. The small sample size is a limitation that should be addressed in future studies, including allowance for subset analyses. Of particular interest is whether habitual performance of yoga would reveal an evolution of changes in sleep architecture, perhaps from acute (restricted to the day of yoga) to chronic (sleep assumes an improved and more stable architecture even between yoga days). From a practical standpoint, longitudinal home monitoring faces potential limitations in the form of missing data (forgotten diary entry or headband falling off in sleep, etc.). Despite these limitations, the opportunities for external validity as well as recognition of sensitive methods to characterize architecture are important advantages. With the increasing availability of home sleep monitoring, longitudinal studies of sleep in the “real world” will provide key insights into our understanding of sleep in health and disease.

## Figures and Tables

**Table 1:** Subject characteristics.

Age	35 median (Range 22–43)
Sex	7 males, 6 females
ESS	6 median (Range 2–10)
Bikram experience	4 experienced, 9 novice
Nights of sleep data	13 median (Range 9–14)
Number of Bikram days	4 median (Range 2–11)
PSQI	8 median (Range 3–14)
Chronotype score	50 median (Range 41–70)
